# The Formation of the Elemental Composition of Young Chardonnay Wine Through Fining with Organic Fining Agents

**DOI:** 10.3390/molecules31183199

**Published:** 2026-09-10

**Authors:** Aleksey Abakumov, Zaual Temerdashev, Evgeniy Gipich, Olga Scheludko

**Affiliations:** 1 Analytical Chemistry Department, Faculty of Chemistry and High Technologies, Kuban State University, Krasnodar 350040, Russia; temza@kubsu.ru (Z.T.); jon.gipich@mail.ru (E.G.); 2 North Caucasian Federal Research Center of Horticulture, Viticulture, Wine-Making, Krasnodar 350072, Russia; scheludcko.olga@yandex.ru

**Keywords:** elemental “image”, wine, wine fining, chemometric, geographical origin

## Abstract

The paper shows the effect of organic fining agents on the formation of the elemental composition of young white wine of the Chardonnay variety. Substances of plant and animal origin and their mixtures were used as fining agents in the clarification of wines. The results were compared with clarification with activated calcium bentonite. In the untreated young wines from different areas of grape cultivation the concentrations of macroelements were 707–837 mg/L; minor elements—5.05–9.67 mg/L; microelements—0.033–0.058 mg/L; and rare earth elements (REEs)—0.001–1.350 µg/L. It was noted that organic agents have a smaller effect on the mineral composition of wine. The content of macroelements in wine decreased, regardless of the fining agent used (*p* < 0.01). After treatment with organic agents, the concentrations of Ti, Sr, Na and Ca increased, while the concentrations of Cu, Zr, Mo, Cs, Ba, W, Fe, Rb and K decreased. No increase in the REE content was observed. A moderate correlation (Pearson r = 0.69, 95% CI: 0.44–0.85, n = 30, *p* < 0.0001) was established between the applied concentration of Na in wine and its concentration in the fining agent. Despite changes, the elemental “image” of wine allows us to correctly determine the geographical origin of the drink. The most reliable markers were Li and Mn.

## 1. Introduction

Each wine variety has a specific set of physical, chemical, and organoleptic characteristics. The variability in the properties of wines within a variety largely depends on specific terroir conditions. [1]. Terroir includes climatic, chemical, and biological environmental factors that interact with viticulture and winemaking technologies, affecting the quality of wine. [2,3,4]. In many countries, researchers can distinguish wines by their region of origin based on information about the migration of elements from rocks to soil, grapes and then to wine [5,6].

When determining the varietal and geographical origin of wine, the main limitation is the representativeness of the choice of elements or compounds in wine and grape samples [7]. The limiting factor in wine authentication is the technological processes used, which can significantly affect the composition of wine and the relationship between wine and soil [8]. Wine fining (stabilization and clarification of wine) is one of the most important stages that affects the quality of the final product. This stage ensures the stable transparency of the drink during storage, eliminating the formation of various types of turbidity and preventing wine aging. Fining agents are often added to wine at this stage. These are substances of various natures, both organic (pea protein, gelatin, etc.) and inorganic (bentonite clays, etc.), which contribute to the removal of colloidal particles that cause wine turbidity. The effects of adding fining agents to wine are mainly considered in terms of reducing the content of proteins, amino acids, polyphenols, and aromatic compounds that affect the quality of the beverage [9,10,11]. At the same time, when wine is clarified with fining agents, there is also a significant change in the elemental composition, which can affect the flavor and significantly complicate the determination of the geographical origin of the finished product [12].

One of the most common fining agents is bentonite clay [7,13,14]. Bentonite suspensions have high adsorption properties due to the high content of colloidal fractions, including amorphous silica [15]. Bentonites have proven to be a high-quality fining agent due to their ability to precipitate unstable forms of compounds [16,17]. However, due to the structure and mechanism of their action, the use of bentonites affects the elemental composition of wine [18,19,20].

Bentonite (montmorillonite) is a layered aluminosilicate mineral with the formula (Na,Ca)_0.3_(Al,Mg)_2_Si_4_O_10_(OH)_2_·nH_2_O, consisting of alternating layers of SiO_4_ tetrahedra and AlO_6_ octahedra, which create a negatively charged surface [21]. When added to wine, bentonite swells and develops a negatively charged surface on which positively charged wine proteins are adsorbed (at pH 3–4) [21]. At the same time, positive ions (Ca^2+^, K^+^, Na^+^) in the electric double layer of clay are replaced by other cations from the solution, and at an acidic pH, H^+^ ions penetrate the mineral structure of wine, breaking Al-O-Si bonds [22]. The Al contained in bentonite clay can partially dissolve at an acidic pH in wine [18]. The released Al remains in the wine solution without precipitating during filtration, leading to a 2–3-fold increase in Al concentration [18].

The authors of [18] indicated an increase in concentrations of Li, Be, Na, and Mg and a decrease in B, K, Cu, Zn and Rb during bentonite treatment, and in [19] there is an increase in concentrations of trace elements in treated wines. In addition, bentonite treatment can significantly affect the content of rare earth elements (REEs), which complicates the use of REE-based wine identification models [20].

Recently, winemakers have been considering the possibility of using organic fining agents: wheat proteins (gluten, prolamins) [23,24], beans (peas, soy, lentils) [25], potatoes (patatin) [26,27], seaweed (spirulina) [28], and grapes (grape seed flour) [29]. The reason for this is that the effect of organic fining agents on wine is considered to be milder and more selective compared to bentonite [30,31].

Treatment with organic fining agents (tannins, plant proteins, gelatin, and chitosan) affects the elemental composition of wine through electrostatic interactions with wine colloids [24]. Tannin-containing agents retain negatively charged phenolic groups at pH 3–4, which are electrostatically attracted to positively charged wine proteins and form hydrogen bonds with polar protein residues. As a result, tannin–protein complexes are precipitated and removed during filtration, without introducing external elements [32]. Plant proteins contain hydrophobic amino acid residues that interact with hydrophobic parts of wine proteins, causing protein aggregation and neutralization of charges [33]. Chitosan is a glucosamine-based biopolymer. At the pH of wine, it protonates its amino groups (-NH_3_^+^), leading to a positive charge, which electrostatically attracts the negatively charged colloids in wine and forms polyelectrolyte complexes that connect small particles [34]. Thus, they extract undesirable components from wine while preserving the qualities inherent in wine. In addition, the use of organic agents is considered a more environmentally friendly approach. Researchers are looking for alternative fining agents that would be effective not only in wine fining, but also in eliminating risks for people with specific nutritional requirements (allergic reactions to animal proteins, etc.). Vegetable proteins can be a worthy alternative to bentonites in this case [26,35,36,37]. Separately, grape seed proteins can be distinguished. They are endogenous components of wine, which reduces the risk of allergic reactions [29].

Along with the prospects and effectiveness of using organic fining agents as an alternative method for wine fining, there are limitations to their use [38,39]. The authors of [40] pointed out the influence of various fining agents on the color, phenolic content, aroma, and sensory properties of red and white wines. On the contrary, [41] showed a slight effect of various organic fining agents—tannin, pea protein, chitosan, gelatin and other fining agents and their mixtures—on the elemental composition and identification properties of Cabernet Sauvignon wines. Although the effect of using organic fining agents has been well-studied in red wines, their effect on white wines remains unclear [41]. For example, in red wines, polyphenolic compounds (tannins, anthocyanins, and flavanols) form a polyelectrolyte matrix that naturally precipitates undesirable particles, including metal complexes, through ligand–metal interaction mechanisms and electrostatic interactions [42]. Red wine proteins are already associated with tannins in the form of stable complexes. Therefore, the added fining agents effectively interact with this formed polyphenol network, providing the predictable removal of proteins, oxidizing agents, and metals [43].

In contrast, white wines are characterized by different conditions. They contain 5–10 times fewer polyphenolic compounds than red wines. This excludes the presence of a developed tannin matrix and a natural pollutant precipitation system. [44]. White wines are characterized by significantly higher acidity, which changes the local charges of functional groups in fining agents, reducing the predictability of their effect [45]. White wine proteins remain predominantly in a free form rather than as complexes with polyphenols and therefore are less protected from the destructive effects of fining agents. As a result, we hypothesize that the elemental composition of white wine may change less predictably after treatment with organic fining agents than in red wine. At the same time, the organic components of white wines, which determine their flavor profile, are more sensitive to the effects of these fining agents due to the lack of polyphenol protection. It can be concluded that the limited knowledge of the possible positive and negative effects of using organic fining agents requires further careful study of their effects on different wine types, as well as their effect on composition, consumer properties, and wine safety.

The purpose of this study was to conduct a comprehensive assessment of the effects of various organic fining agents and activated calcium bentonite on the quality, safety, and identification characteristics of white Chardonnay wine. We previously discussed some issues related to the influence of various fining agents on the quality and identification characteristics of red wines [41]. The present work is a logical continuation of these studies on white wines made from the Chardonnay grape variety.

## 2. Results and Discussion

### 2.1. Validation of the Research

To ensure the reliability, accuracy, and precision of the data obtained, all analytical measurements were subjected to strict quality control. The linearity of calibration dependences was confirmed for each measurement session using the coefficient of determination. R^2^ > 0.995. The accuracy of determining the elements in the studied objects was controlled by analyzing certified reference samples (Supplementary Table S1) and by the spike method (Supplementary Table S2). The criterion for acceptability in the spike method was recovery values at a level of 90–110%.

Precision was assessed by relative standard deviation (RSD). Every tenth sample was analyzed in duplicate, with RSD values above 10% acting as a signal for re-measuring or excluding a point from the dataset. To compensate for instrument drift during long-term analysis series, the instrument was automatically recalibrated after every 20 samples.

To take into account the matrix effects and minimize their effect on the results of the wine analysis, a tenfold dilution (1:10 by volume) with 2% nitric acid was used. This, combined with the use of external calibration curves, made it possible to reduce the concentrations of organic acids, sugars, and alcohols to levels that preclude plasma disruption [41]. The correctness of this approach was confirmed by testing samples using the spike method over the entire operating concentration range. The studied objects were analyzed simultaneously using inductively coupled plasma atomic emission spectrometry (ICP-AES) for Al, Ba, Be, Ca, Cu, Fe, K, Mg, Mn, Na, Ni, Rb, Sr, and Zn, and inductively coupled plasma mass spectrometry (ICP-MS) for Cd, Ce, Co, Cs, Dy, Er, Eu, Gd, Ho, La, Li, Lu, Mo, Nd, Pb, Pr, Sm, Tb, Ti, Tm, V, W and Y. This approach minimized spectral interference and ensured maximum detection sensitivity.

### 2.2. Elemental Analysis of Fining Agents

**Figure 1:**
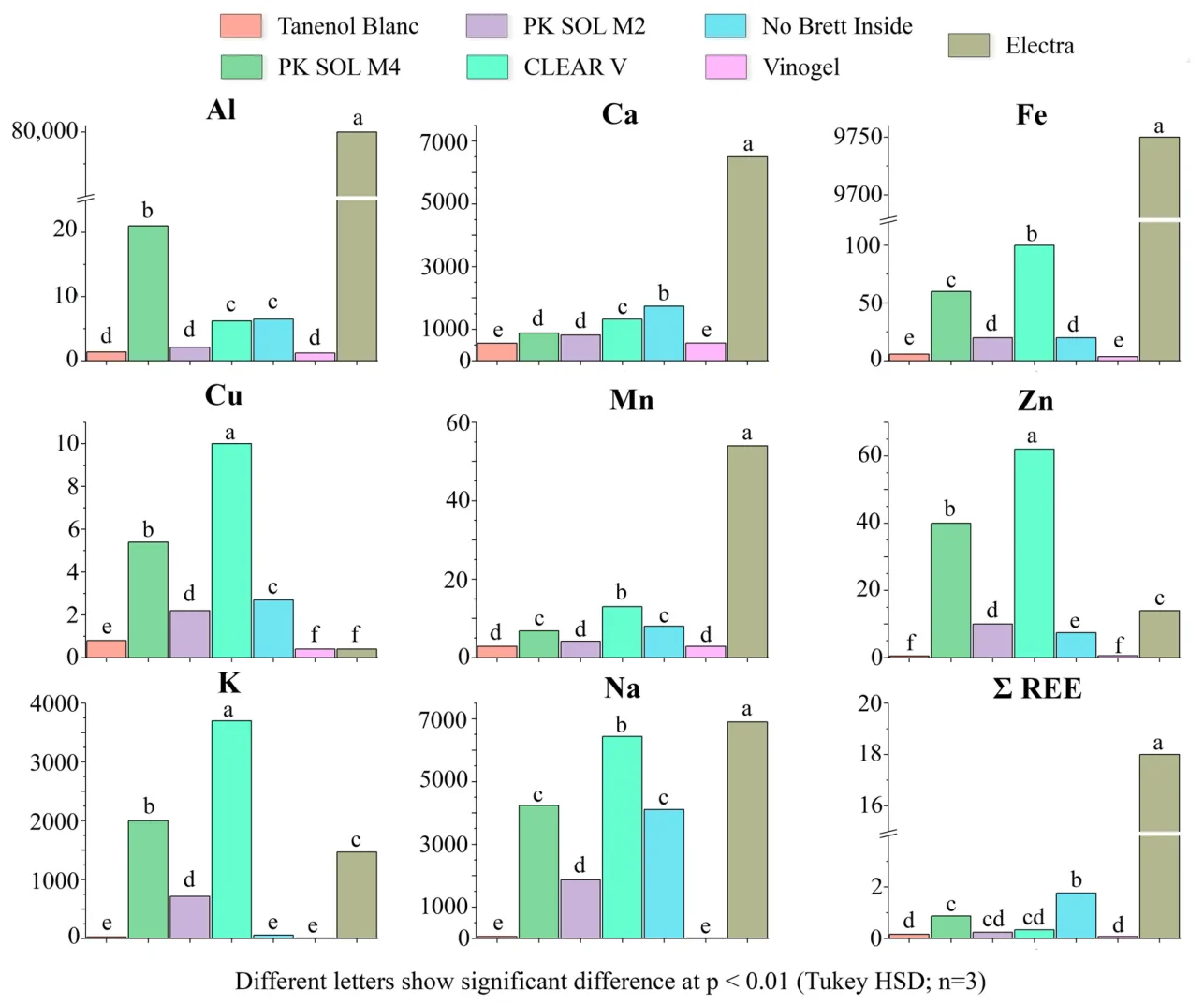
The content of certain elements in fining agents, mg/kg.

As expected, higher concentrations of almost all elements (especially Al, Ca, Na, and Fe) were observed in Electra bentonite clay. (Figure 1). This is due to the mineral nature of bentonite, which is based on sodium–calcium montmorillonite (basal reflection (001) at 15.04 Å), with an admixture of quartz (up to 3%) [8].

The results of one-way analysis of variance (one-way ANOVA) showed statistically significant differences (*p* < 0.01) between the concentrations of most elements in the studied fining agents, except for Li and Be, whose content in organic agents was the lowest. It should be noted that PK SOL M4 and No Brett Inside had the highest concentrations of rare earth elements (REEs) of all the organic fining agents.

### 2.3. Formation of the Elemental Composition of Wine After Clarifying with Fining Agents

After wine fining, the total content of macro- (Ca, K, Na and Mg), minor (Al, Ba, Cu, Fe, Mn, Ni, Rb, Sr, Ti and Zn), and microelements (Be, Cd, Co, Cs, Li, Mo, Pb, V, W and Zr), as well as REEs (Y, La, Ce, Pr, Nd, Sm, Eu, Gd, Tb, Dy, Ho, Er, Tm, Yb and Lu) changed. These changes are shown in Table 1. The dataset with the concentrations of all elements is presented in Supplementary Table S3.

The untreated wines have different elemental compositions depending on their growing area. The concentration of trace elements ranged from 707 to 837 mg/L for macroelements; 5.05 to 9.67 mg/L for minor elements; 0.033 to 0.058 mg/L for microelements and 0.0 to 1.35 µg/L for REEs. The highest concentrations of macro-, micro-, and rare earth elements were observed in wine from Vinnaya derevnya, while the highest minor amounts were found in wines from Vinogradny. No REEs were found in untreated Varvarovka and Vinogradny wines.

Significant differences (*p* < 0.01) in the elemental composition of young wines were observed after treatment, which differed between the various fining agents. However, these changes were not dependent on the wine growing area. A statistically significant (*p* < 0.01) decrease in the content of macroelements after wine fining was established. This fact can be explained by the precipitation of tartaric and tannic acid salts during fermentation. The concentrations of minor elements changed insignificantly (<5%) after treatment with Electra and Tanenol Blanc. The effect of Vinogel on the content of minor elements was also insignificant, with the exception of samples from Varvarovka and Vinogradny. The PK SOL M4 and PK SOL M2 reduced the concentration of minor elements by 15–20%, while “No Brett Inside” and Clear V obtained reductions of 20–30%.

All samples of organic agents reduce the content of trace elements in wine, but the degree of reduction depends on the specific agent. The strongest effect was demonstrated by No Brett Inside, which reduced the total content of microelements by 10–30%. Bentonite fining significantly increased the concentration of microelements in wine by 10–30% compared to organic agents.

In all untreated wines, Y, La and Ce were reliably determined. The other REEs in the untreated wine samples, despite the relatively high sensitivity of ICP-MS analysis [46,47], were not detected. After treatment with organic agents, the REEs content in wine decreased, and after bentonite fining, their concentration increased by 1.0–4.5 µg/L.

### 2.4. Analysis of the Interaction Between Wines and Fining Agents

The effect of fining agents on the elemental composition of wine was studied to assess compliance with international and national safety standards, as well as the nature of their effects on physicochemical stability and organoleptic properties. The International Organization of Vine and Wine (OIV) have established maximum permissible concentrations for a number of elements, including As, Cd, Pb, Cu and Zn [48]. Also, some countries have introduced stricter or additional restrictions in their laws [48,49,50]. The analysis of the content of elements in wines showed that none of the studied samples, before or after treatment with various fining agents, exceeded the OIV standards (Table 2).

**Figure 2:**
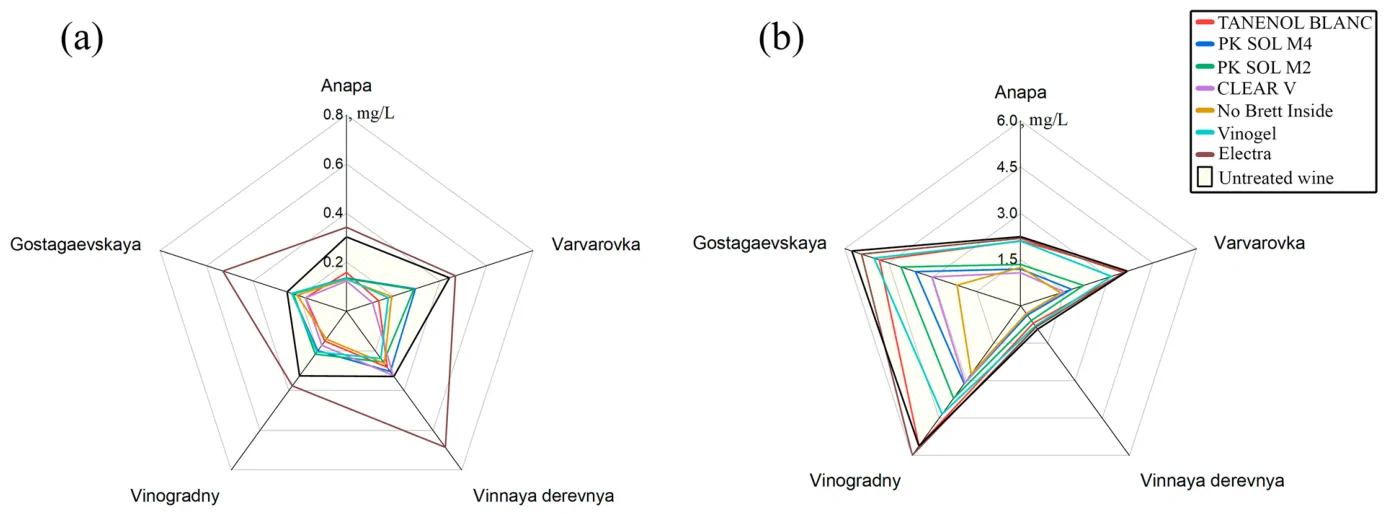
The effect of wine fining on Al (**a**) and Fe (**b**) contents in Chardonnay wine.

The concentrations of Ti, Sr, Na and Ca in organic-treated wines were higher than the initial contents, as was also observed for Cabernet Sauvignon wines [41]. A decrease in the concentrations of Cu, Zr, Mo, Cs, Ba, W, Fe, Rb and K was observed during treatment with both organic agents and bentonite. However, for these specific elements, the decrease was usually more pronounced when treated with bentonite than when using organic agents. For example, the concentration of Cu decreased by 5–30% with organic agents, compared to 10–65% when treated with bentonite. When bentonite was used, the concentration of Al in wine increased, whereas organic agents decreased it (Figure 2a). It can be noted that a notable decrease in Fe concentration is observed after fining with No Brett Inside and Clear V (Figure 2b).

An increase in Al content (>5 mg/L) can negatively affect the organoleptic properties of wine, giving it a metallic taste and an off-odor or causing turbidity (>10 mg/L) [51]. Bentonite is one of the potential sources of Al input into wine. However, in the resulting wine samples, the concentration of Al was at a level that did not affect the taste or aromatic characteristics of the wine.

**Figure 3:**
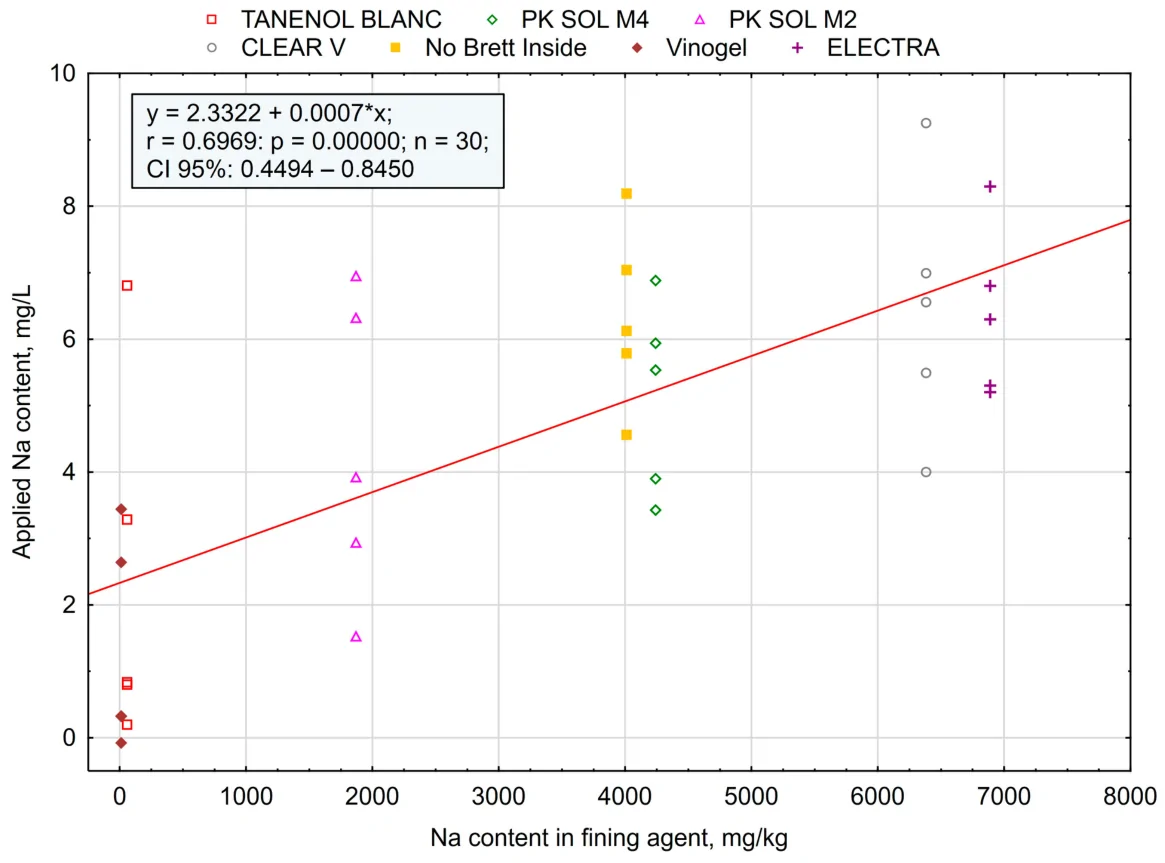
Correlation between the content of Na added to the wine and the content of Na in the fining agent.

When clarifying wine, the Na content varies greatly. This is primarily due to the bentonite mechanism of cation exchange, in which Na^+^ ions are replaced while binding proteins in wine [18]. It has been established that organic fining agents contribute concentrations of Na comparable to bentonite and sometimes even higher. Figure 3 shows the correlation between the Na content in the fining agent and the final Na concentration in wine. Correlation analysis revealed a strong positive relationship (Pearson r = 0.69, 95% CI: 0.44–0.85, n = 30, *p* < 0.0001) between the Na content in the fining agent and the final Na concentration in treated wine. This means that fining agents are a source of Na in the wine. The more Na in the agent, the more Na will be added to the wine.

The treatment of young wine with organic agents reduces the concentration of Cu and Fe, reducing the risk of “copper” and “iron” turbidity developing and improving the oxidative stability of wine [52]. Bentonite reduced the concentration of Cu, but it had little effect on Fe content. The high K content affects the possibility of crystal turbidity and increases the risk of the formation of tartaric precipitations [53]. Therefore, the observed decrease in K concentration when using any fining agent is a positive factor. At the same time, organic and inorganic fining agents can increase Ca content. Increased concentrations of Ca in wines (>100 mg/L) facilitate the formation of insoluble calcium tartrate, crystalline and colloidal turbidity [18]. The concentration of Ca in the treated young wines did not reach values leading to a risk of crystalline turbidity [54]. Thus, fining agents can serve not only as an effective clarifier of wine, but also as a tool for correcting the mineral composition of wine.

### 2.5. The Influence of Fining Agents of Various Natures on the Geographical Origin Identification of Young Wines

Analysis of the composition of wine treated with various fining agents shows that these agents have a multidirectional effect on wine’s elemental composition, which may affect the relationship between the elemental composition of wine, grapes and soil. This means that when establishing marker elements, it is necessary to take into account the contribution of various fining agents to the formation of the elemental composition of wine.

Discriminant analysis assessed the effect of fining agents on the relationships in the “soil–grape–wine” system, and also identified marker elements for establishing geographical origin. An important note should be made regarding the design of the discriminant analysis, as the samples form a nested structure. There are three independent samples for each of the five regions. From each of these three samples, seven more samples were subsequently obtained by clarification with various agents. At first glance, this data structure represents statistical complexity (pseudo-repetition), but it also demonstrates the key result of this study. This consists of the fact that, despite significant changes in the elemental composition caused by various fining agents, samples from the same vineyard form groups, which indicates the preservation of the geographical identity of the wine.

The predictors (independent variables) of the model are concentrations of elements in wines. The grouping (dependent) variable is the area of grape cultivation. When constructing the discrimination model, a step-by-step exclusion method was used. This applies the automatic exclusion of variables based on F-remove statistics and allows for an interactive viewing of the procedure for excluding variables. As, Cd and REEs were not included in the model because of their low concentrations. To evaluate the classification properties of the identification model, the dataset was divided into training and test datasets during its construction. The test sample included one random biological sample of wine from each category (5 regions × 7 fining agents + 5 untreated wines = 40 samples). The remaining 80 samples were placed in the training set. Note also that the splitting of the training and test datasets was performed at the level of independent initial fermentations. Thus, samples obtained as a result of the same fermentation were not distributed between the training and test datasets. This approach was applied to preserve the original data structure and ensure high-quality training of the model. Thus, the distribution of samples between training and test datasets was approximately 70% and 30%, respectively. According to the results of the analysis, six significant predictors remained in the model (Table 3). The small value of Wilks’s lambda (0.0000) in the upper part of the table indicates successful discrimination, confirming the formation of clusters based on the regional origin of wines.

**Figure 4:**
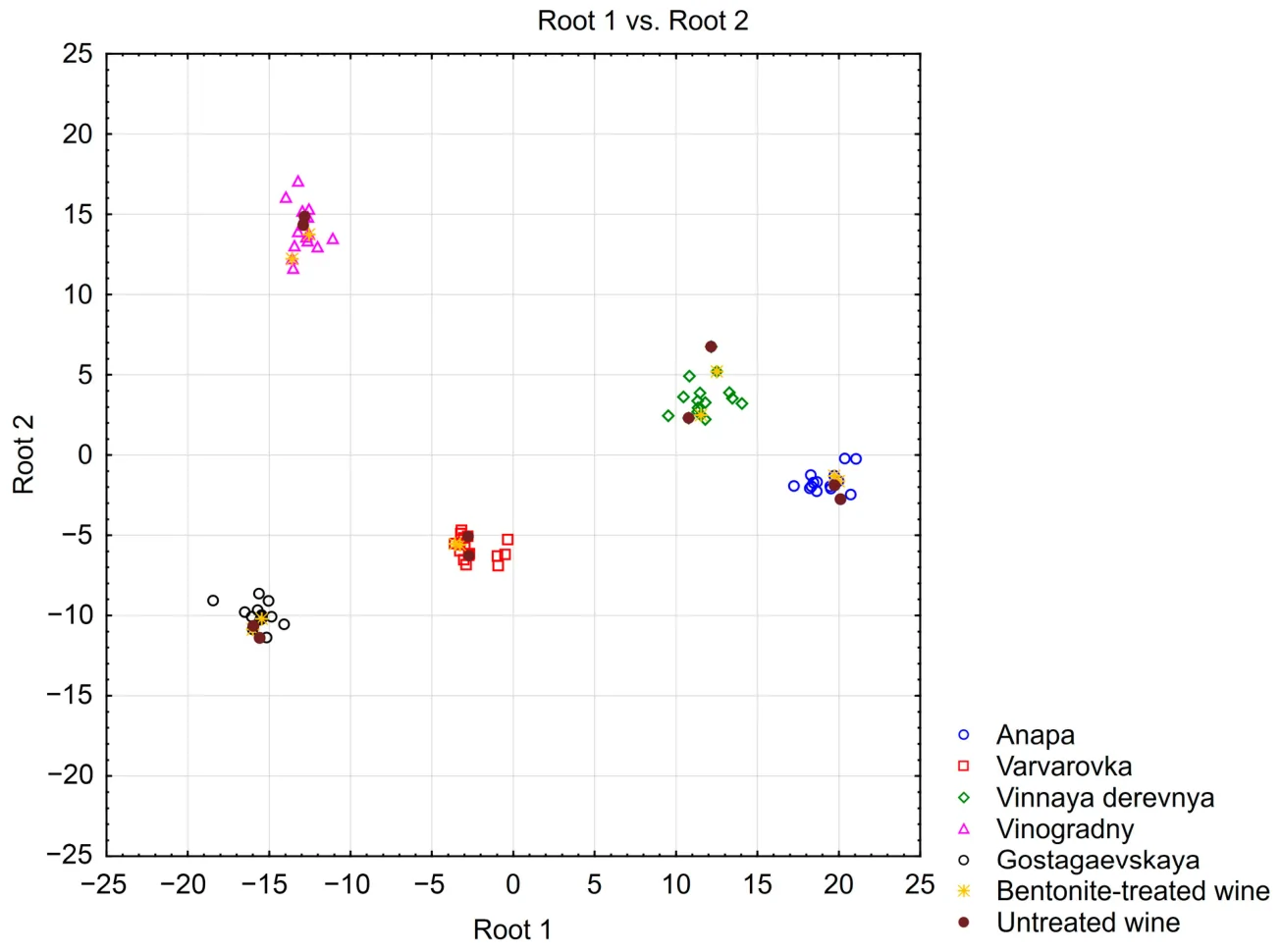
Scatterplot of canonical values of untreated, bentonite-treated and organic-treated wine samples in training dataset.

The scatterplot of canonical values is a graphical illustration of the presence of a regional cluster structure (Figure 4). It allows you to transfer objects from a multidimensional space into a space of a smaller dimension—to a plane—while maintaining the distances between objects. This scatterplot can be used to judge the uniformity and degree of similarity between groups by using distances based on the principle that the smaller the distance, the greater the similarity.

It can be seen that the samples from each region are localized in their “own” part of the plane, forming clusters (Figure 4). The high density of treated young wines of the same growing area indicates the degree of similarity between the compositions of these wines. According to the results of the discriminant analysis, classification functions were obtained (Supplementary Table S4). The classification accuracy was 100% for both the training and the test datasets. This suggests that, despite significant changes in the concentration of individual elements during wine fining, the final elemental composition reflects the connection between wine and its terroir. It can be argued that Li and Mn are potentially the most reliable markers. These elements are generally recognized as markers of terroir in many studies, due to the high correlation between the concentrations of Li and Mn in wine and the geochemical characteristics of the soil [6].

### 2.6. Limitations of the Research

This study was performed on young Chardonnay wine from a single harvest (2022 year) and a single geographical region. The results cannot be generalized to other grape varieties, different harvests, or wines from other wine regions without additional research. Seven commercially available fining agents were tested. Other clarification methods, such as cross-microfiltration, centrifugation, or other fining agents, were not evaluated and may produce different results. At the same time, the formation of the elemental composition was studied only during the wine fining stage. Changes in elemental composition during exposure and storage were not considered.

Although the concentrations of the elements were measured with high precision, no organoleptic evaluation was performed by trained tasters. Changes in the elemental composition of wines have been identified, but there is no direct evidence that these changes can be detected by humans through sensory assessment.

Despite these limitations, the data obtained provides a reliable methodological basis for the practical implementation of a geographical origin protection system for wines. In the near future, it is planned to create an open regional database of elemental “fingerprints” for the wines of Krasnodar Territory.

## 3. Materials and Methods

### 3.1. Objects of Research

Six substances of different nature were studied as organic fining agents: Tanenol Blanc (Enartis, Piemont, Italy)—gallic tannin; fining mixtures PK SOL M2 (Perdomini-IOC, San Martino Buon Albergo, Italy)—PVPP + chitosan + pea protein and PK SOL M4 (Perdomini-IOC, San Martino Buon Albergo, Italy)—chitosan+ pea protein + silica gel; Clear V (Perdomini-IOC, San Martino Buon Albergo, Italy)—pea protein; No Brett Inside (Lallemand, Montreal, QC, Canada)—chitosan; Vinogel (Delo Vkusa, Moscow, Russia)—gelatin; and the activated calcium bentonite Electra (Martin Vialatte, Magenta France). More detailed information about the fining agents is provided in Supplementary Table S5.

Wines made from Chardonnay grapes were used in research on fining with various fining agents. The grapes were grown on five vineyards in the Anapa district of the Krasnodar Territory, Russia, using the same Kober 5BB rootstock, but different types of soil (Table 4).

### 3.2. Winemaking Scheme

The grapes were harvested upon reaching the stage of technical maturity. Wines were prepared from Chardonnay grapes of the 2022 harvest year. For each of the five vineyards, three independent fermentations were carried out using the same grape variety and harvest period. Each fermentation represents an independent full winemaking cycle under identical conditions (temperature, fermentation duration, yeast culture, etc.) This allowed us to consider micro-variability in the wine-making process. Thus, the total number of untreated young wines was 15 (5 vineyards × 3 independent fermentations).

Processing was carried out in accordance with the recommendations [55]. Crushing was carried out on roller crushers (DMCSI Grifo, Flussio, Italy) with separation of ridges. Next, the crushed grapes were pressed using a pneumatic press (Busher Vaslin, Chalonnes-sur-Loire, France). No more than 7 hL of juice per ton of grapes was used to produce dry white wine. The pressed and drained juice was sulfitized with the preparation “ Ultrasulph S” (Perdomini-IOC, San Martino Buon Albergo, Italy) at a rate of 80–150 mg of SiO_2_ per 1 kg of processed grapes, which was further clarified by settling for 12 h at temperature of 8 °C. Then, the juice was removed from the sludge and fermented in fermentation tanks. An active pure culture of dry yeast (Lallemand Lalvin EC 1118 (Lallemand, Montreal, QC, Canada)) was added at doses recommended by manufacturers (30 g/hL). The fermentation process was carried out for 7 days at a temperature not exceeding 18 °C. Upon completion of sugar fermentation and separation of yeast sediment, the wine was again treated with “Ultrasulph S” at a rate of 50 mg/L of SO_2_. The produced wine is characterized as a young wine. All wine samples were stored at 5 °C in tightly closed, fully filled, green glass bottles in a dark place, excluding any possible influence of external factors. The main physico-chemical properties of the studied wine samples are given in [56].

### 3.3. Procedure for Clarification and Stabilization of Young Wines

Each of the 15 young wines was divided into eight parts, each measuring 0.5 L. Seven of these were treated with fining agents, while one sample was used as a control. Thus, the scheme of the experiment was: 5 vineyards × 3 independent fermentations × 8 treating options = 120 individual wine samples. Each sample was analyzed in three technical replicates using ICP-AES and ICP-MS methods, and the mean value of the element concentrations was taken as the result. The wine fining was carried out under controlled conditions, excluding exposure to light, oxygen in the air, and temperature fluctuations. Taking into account the manufacturer’s recommendations [57,58,59], the optimal dosages of organic fining agents were selected. The wine fining using bentonite was carried out using well-known techniques [60]. After applying the fining agents to the wine, it was aged for 24 h at a temperature of 10–15 °C in a dark place. During the contact time, the samples were periodically mixed to improve the interaction between the fining agents and the wine. Then, filtration was carried out using a Super-Jet Wine Filter (Buon Vino, ON, Canada) with a pore size of 2 microns. The samples were stored in tightly closed and fully filled glass bottles in a dark place at a constant temperature of 20 ± 2 °C.

Prior to the start of the main study, a series of trial treatments were conducted to determine the optimal dosage for each fining agent. Each agent was tested on samples of young Chardonnay wine (volume 100 mL) at dosages recommended by the manufacturers. During the treatments, the following parameters were evaluated: the rate of precipitation of suspended particles (visually after 2, 4 and 6 h), whether transparency was achieved (transmission of light through the sample), and the presence of excess sediment. Based on the results, an optimal dosage was selected for each fining agent to ensure the effectiveness of precipitation while maintaining the quality characteristics of wine. The selected dosages were: Tanenol Blanc—50 mg/L; PK SOL M2—250 mg/L; PK SOL M4—150 mg/L; Clear V—200 mg/L; No Brett Inside—60 mg/L; Vinogel—30 mg/L; and Electra—300 mg/L.

### 3.4. Preparation of Fining Agents for Element Analysis

Preparation of the bentonite sample for elemental analysis was carried out according to the method described in [7]. The preparation of organic fining agents for elemental analysis was carried out using the acid mineralization in the Ethos 1 (Milestone, Milan, Italy) microwave decomposition system. A total of 0.5 g of the sample was placed in an autoclave, and 5 mL of concentrated HNO_3_, 3 mL of H_2_O_2_, and 2 mL H_2_O were added. The sample digestion process includes two stages: gradual heating of the mixture to 200 °C for 25 min, followed by exposure at this temperature for 5 min. After completion of the decomposition program, the autoclaves were cooled to temperatures below 40 °C to prevent loss of volatile elements. Then, the contents were transferred quantitatively to a 50 mL measuring flask and brought up to the mark with deionized water with a maximum resistivity of 18.2 MΩ·cm. The completeness of the decomposition of fining agents and the accuracy of the results of the element determination were assessed using standard samples of corn flour (INCT-CF-3) and soy flour (INCT—SBF-4) (Supplementary Table S1). The wines were prepared for analysis by pre-diluting them 15 times with 2% nitric acid, taking into account the recommendations [41].

### 3.5. Elemental Analysis of Wines

The concentrations of elements were quantified using two complementary plasma-based spectrometric techniques: ICP-AES (iCAP 7400, Thermo Scientific, Waltham, MA, USA) for major and minor elements (Al, Ba, Be, Ca, Cu, Fe, K, Mg, Mn, Na, Ni, Rb, Sr, Zn) and ICP-MS (ICAP-RQ, Thermo Scientific, Waltham, MA, USA) for trace and rare earth elements (Cd, Ce, Co, Cs, Dy, Er, Eu, Gd, Ho, La, Li, Lu, Mo, Nd, Pb, Pr, Sm, Tb, Ti, Tm, V, W, Y, Yb, Zr) [61].

Single-element reference standards (1000 mg/L; Inorganic Ventures, Christiansburg, VA, USA) were used to prepare multi-point calibration curves (R^2^ > 0.995). Quality assurance included duplicate measurements (10% of samples), matrix blanks, and recovery tests on spiked samples (target: 90–110%). Instrumental parameters are detailed in Supplementary Table S6.

### 3.6. Statistical Analysis

The estimation of statistically significant differences between wine samples was carried out using one-way analysis of variance (one-way ANOVA, *p* < 0.01) with the post hoc Tukey honestly significant difference (Tukey HSD) test. One-way ANOVA and Tukey HSD tests were performed in R using the multcomp (package version 1.4–28) [62]. The degree of similarity and differences between treated and untreated wines in terms of concentrations of analytes were assessed using discriminant analysis. Using discriminant analysis, the elements that contribute most to the separation of samples based on their elemental composition were identified. Discriminant analysis was performed using STATISTICA (v. 14.0.0.15) (Tibco, Palo Alto, CA, USA).

## 4. Conclusions

The influence of organic fining agents on the formation of the elemental composition of young white Chardonnay wine was analyzed. Untreated young Chardonnay wines from five different vineyards on the Black Sea coast differed in their elemental compositions. After treatment with fining agents of organic and inorganic nature, it was found that the organic agents had less effect on the mineral composition of wine. When treated with organic agents, an increase in the concentrations of Ti, Sr, Na and Ca was observed, while a decrease in Cu, Zr, Mo, Cs, Ba, W and Fe, as well as Rb and K, was also observed. No increase in REEs was established.

The results of the studies show that fining agents of organic origin can be an effective and safe alternative to bentonite in the production of white wines, with minimal effects on their mineral composition. It is also important that treatment with organic fining agents preserves the elemental composition of wine, reflecting its geographical origin. Discriminant analysis showed the preservation of their cluster structure after treatment with organic fining agents. It can be argued that Li and Mn are potentially the most reliable markers. Nevertheless, research is required to study the transformations of organic components in wine when treated with organic agents to confirm or disprove the preservation of its quality characteristics.

It is worth noting that this study is only based on wine from the 2022 harvest, which limits the generalizability of the results. Further studies on wine from two to three consecutive harvest years with different climatic conditions are needed to confirm and summarize the findings. This will make it possible to assess the stability of the obtained results to interannual climatic and soil variability and determine whether the observed patterns are robust and reproducible regardless of the harvest year.

## Figures and Tables

**Table 1:** Total content of elements in wine before and after treatment with fining agents.

Total Content of Elements	Untreated Wine	Wine Treated with Fining Agent						
		Tanenol Blanc	PK SOL M4	PK SOL M2	Clear V	No Brett Inside	Vinogel	Electra
**Gostagaevskaya**								
macro-, mg/L	794 ^a^	716 ^b^	711 ^b^	726 ^b^	703 ^b^	687 ^b^	703 ^b^	720 ^b^
minor, mg/L	9.10 ^a^	7.99 ^bc^	7.41 ^c^	7.28 ^c^	6.36 ^d^	5.46 ^e^	8.58 ^ab^	8.72 ^ab^
micro-, mg/L	0.054 ^b^	0.050 ^b^	0.045 ^bc^	0.047 ^bc^	0.045 ^bc^	0.039 ^c^	0.044 ^bc^	0.063 ^a^
REE, µg/L	0.438 ^b^	0.038 ^c^	0.031 ^c^	0.029 ^c^	0.025 ^c^	0.037 ^c^	0.018 ^d^	4.71 ^a^
**Vinogradny**								
macro-, mg/L	757 ^a^	640 ^b^	636 ^b^	606 ^b^	625 ^b^	622 ^b^	645 ^b^	657 ^b^
minor, mg/L	9.67 ^a^	9.62 ^a^	7.21 ^cd^	7.81 ^bc^	7.04 ^cd^	6.63 ^cd^	8.39 ^b^	10.3 ^a^
micro-, mg/L	0.045 ^b^	0.040 ^cd^	0.041 ^bc^	0.040 ^cd^	0.040 ^cd^	0.036 ^d^	0.041 ^bc^	0.061 ^a^
REE, µg/L	0 ^b^	0 ^b^	0 ^b^	0 ^b^	0 ^b^	0 ^b^	0 ^b^	2.59 ^a^
**Anapa**								
macro-, mg/L	707 ^a^	641 ^ab^	648 ^ab^	658 ^ab^	633 ^b^	665 ^ab^	653 ^ab^	622 ^b^
minor, mg/L	5.67 ^ab^	5.36 ^ab^	4.76 ^cd^	4.50 ^de^	4.10 ^e^	4.34 ^de^	5.13 ^bc^	5.70 ^a^
micro-, mg/L	0.044 ^b^	0.038 ^cd^	0.041 ^bc^	0.034 ^ef^	0.037 ^de^	0.031 ^f^	0.037 ^de^	0.051 ^a^
REE, µg/L	0.378 ^b^	0.122 ^d^	0.310 ^c^	0.261 ^ef^	0.250 ^ef^	0.288 ^cf^	0.232 ^e^	1.11 ^a^
**Varvarovka**								
macro-, mg/L	804 ^a^	703 ^b^	672 ^b^	711 ^b^	666 ^b^	713 ^b^	688 ^b^	678 ^b^
minor, mg/L	7.36 ^a^	6.93 ^ab^	5.45 ^c^	5.63 ^c^	4.7 ^d^	4.55 ^d^	6.57 ^b^	7.06 ^ab^
micro-, mg/L	0.033 ^b^	0.024 ^c^	0.026 ^c^	0.024 ^c^	0.024 ^c^	0.022 ^c^	0.024 ^c^	0.042 ^a^
REE, µg/L	0 ^b^	0 ^b^	0 ^b^	0 ^b^	0 ^b^	0 ^b^	0 ^b^	5.41 ^a^
**Vinnaya derevnya**								
macro-, mg/L	837 ^a^	704 ^b^	731 ^b^	738 ^b^	699 ^b^	719 ^b^	728 ^b^	724 ^b^
minor, mg/L	5.05 ^b^	4.73 ^b^	4.79 ^b^	4.77 ^b^	4.37 ^c^	4.17 ^c^	4.72 ^b^	5.56 ^a^
micro-, mg/L	0.058 ^b^	0.052 ^c^	0.052 ^c^	0.052 ^c^	0.049 ^c^	0.050 ^dc^	0.054 ^c^	0.078 ^a^
REE, µg/L	1.35 ^b^	0.868 ^cd^	0.816 ^cd^	0.729 ^d^	0.789 ^de^	0.986 ^c^	0.660 ^e^	2.48 ^a^

Note: The values in the table represent the mean of three independent biological replicates (three independent fermentations performed for each vineyard under identical conditions). Different letters in the same row show a statistically significant difference at *p* < 0.01 (Tukey HSD).

**Table 2:** Maximum permissible element contents in wines [48,49,50].

Element	The Standard for the Content of the Elements, mg/L								According to This Research, mg/L		
	OIV	Mexico	Chile	Germany	EU	Canada	Russia	Croatia	Untreated Wine	Bentotine-Treated	Organic-Treated
Ag	n/s	n/s	n/s	n/s	n/s	n/s	n/s	0.1	<LOQ	<LOQ	<LOQ
Al	n/s	n/s	n/s	8.0	n/s	n/s	n/s	10	0.33	0.05	0.20
As	0.2	0.5	0.2	0.1	n/s	0.1	0.2	0.2	<LOQ	<LOQ	<LOQ
Cd	0.01	n/s	0.01	0.01	n/s	n/s	0.03	0.01	0.25 × 10^−3^	0.23 × 10^−3^	0.24 × 10^−3^
Cu	1.0	n/s	1.0	2.0	n/s	1.0	n/s	1.0	0.057	0.043	0.051
Fe	n/s	n/s	n/s	n/s	n/s	n/s	n/s	20	3.6	3.6	2.4
Na	n/s	n/s	n/s	n/s	60	n/s	n/s	20	7.6	14	12
Ni	n/s	n/s	n/s	n/s	n/s	n/s	n/s	0.1	0.17	0.16	0.16
Pb	0.15	0.5	0.15	0.25	0.2	0.2	0.3	0.3	0.030	0.004	0.030
Sn	n/s	n/s	n/s	1.0	n/s	250	n/s	n/s	<LOQ	<LOQ	<LOQ
Zn	5.0	n/s	n/s	5.0	n/s	n/s	n/s	5.00	0.74	0.58	0.67

Note: n/s—there are no standardized values.

**Table 3:** Results of the discriminant analysis of wines.

N = 80	Variables in the Model: 6; Grouping.: Cultivation Area (5 gr.) Wilks Lambda: 0.00000 Approx. F (24.245) = 978.66 *p* < 0.0000					
	Wilks Lambda	Partial Lambda	F-Remove (4.70)	p-Level	Tolerance	1-Tolerance (R-sq.)
Cs	0.005 × 10^−3^	0.022	792.66	0.001 × 10^−11^	0.353	0.647
Cu	0.002 × 10^−3^	0.053	313.83	0.001 × 10^−11^	0.600	0.400
Fe	0.002 × 10^−4^	0.615	10.94	0.001 × 10^−3^	0.590	0.410
Li	0.003 × 10^−3^	0.034	491.34	0.001 × 10^−11^	0.880	0.120
Mn	0.006 × 10^−3^	0.018	931.89	0.001 × 10^−11^	0.901	0.099
Zr	0.003 × 10^−4^	0.426	23.60	0.002 × 10^−9^	0.321	0.679

**Table 4:** Information about the vineyards where grapes are cultivated.

Indicator	Vineyard				
	Vinogradny	Gostagaevskaya	Varvarovka	Vinnaya Derevnya	Anapa
Soil type	Southern chernozem formed on sands and light loams	Rendzina formed on carbonaceous shales	Rendzina	Southern chernozem	Southern carbonate chernozem
Granulometric composition	Light loamy	Heavy loamy	Heavy loamy	Heavy loamy	Medium loamy
Humus, %	1.9–2.6	2.6–2.8	1.8–2.4	1.6–2.0	1.6–2.2
Year of planting	2018	2019	2019	2017	2013
Planting scheme	3.0 × 2.0	3.0 × 1.5	3.0 × 2.0	3.0 × 1.5	3.0 × 1.5
Grapevine shape	Single-shoulder cordon	Single Guyot	Double-shoulder cordon	Single-shoulder cordon	Double-shoulder cordon
Fertilizers	Nutrivant plus (3.0 kg/ha)	Nutrivant plus (3.0 kg/ha); Fungicrops (3–4 L/ha)	EcoRic (4.0 L/ha); Lignohumate (1.0 L/ha)	Raykat (1.0 L/ha); Fungicrops (3–4 L/ha)	Raykat (1.0 L/ha)

## Data Availability

The original contributions presented in this study are included in the article/supplementary material. Further inquiries can be directed to the corresponding author.

## Supplementary Materials

The following supporting information can be downloaded at https://www.mdpi.com/article/10.3390/molecules31183199/s1, Table S1: Assessment of the correctness of applied methods of analysis of fining agents; Table S2: Assessment of the correctness of wine analysis using the spike method; Table S3: The concentrations of elements in wines; Table S4: Classification functions obtained using discriminant analysis; Table S5: Information about fining agents; Table S6: Operational parameters of spectrometers and limits of quantification.
