# Supplementary material for: The Formation of the Elemental Composition of Young Chardonnay Wine Through Fining with Organic Fining Agents
Source: Molecules. 2026 Sep 10;31(18):3199. doi: 10.3390/molecules31183199 (PMC13608984; doi:10.3390/molecules31183199)
Supplement: Supplementary file 1 [file molecules-31-03199-s001.zip › Supplementary Table S1.pdf]

Supplementary Table S1. Assessment of the correctness of applied methods of analysis of fining agents

Element	Corn flour (INCT-CF-3)		Soy flour (INCT-SBF-4)	
	Certified value, mg/kg	Found, mg/kg n=3, P=0.95	Certified value, mg/kg	Found, mg/kg n=3, P=0.95
Al	-	-	45.5±3.7	43±13
B	1.65±0.33	-	39.3±4.0	36±7
Ba	-	-	7.3±0.23	7.6±2.1
Ca	-	-	2467±170	2410
Cu	1.63±0.13	1.6±0.4	14.30±0.46	15±4
Fe	32.0±1.4	30±9	90.8±4.0	92±28
K	3157±119	3200±650	2.423±0.083	25000±5000
Mg	1066±37	1090±250	3005±82	3060±760
Mn	4.98±0.22	5.0±1.5	32.3±1.1	31±9
Mo	152±12	-	5.99±0.35	6.1±1.8
Ni	0.383±0.039	0.40±0.14	3.12±0.18	3.2±1.0
Rb	0.912±0.042	0.89±0.19	31.7±1.7	-
Sr	-	-	9.32±0.46	10±2
Zn	20.09±0.76	22±6	52.3±1.3	53±13
