# Supplementary material for: The Formation of the Elemental Composition of Young Chardonnay Wine Through Fining with Organic Fining Agents
Source: Molecules. 2026 Sep 10;31(18):3199. doi: 10.3390/molecules31183199 (PMC13608984; doi:10.3390/molecules31183199)
Supplement: Supplementary file 1 [file molecules-31-03199-s001.zip › Supplementary Table S2.pdf]

Supplementary Table S2. Assessment of the correctness of wine analysis using the spike method

Element	Concentration, µg/L			
	Sample	Spike	Sample + Spike	**R, %**
**Al**	302	300	590	96.0
	440	300	762	105.0
	328	300	630	100.6
**K**	581022	500000	1105721	104.3
	674795	500000	1190522	102.3
	687793	500000	1199021	101.6
**Ca**	65176	50000	113978	98.2
	40813	50000	92862	105.0
	64483	50000	118052	105.5
**Ni**	112	100	214	101.8
	126	100	239	110.3
	169	100	265	97.6
**Sr**	413	400	825	102.9
	600	400	963	93.8
	332	400	778	113.9
**Zn**	513	500	987	94.9
	644	500	1156	101.9
	787	500	1323	104.6
**V**	4.51	5	9.76	105.5
	2.48	2.5	5.08	104.0
	5.33	3	8.53	103.8
**W**	1.00	1.00	2.02	101.8
	3.28	2.00	5.31	100.9
	2.84	2.00	4.99	105.2
**La**	0.103	0.100	0.205	101.9
	0.000	0.100	0.099	99.0
	0.260	0.200	0.461	100.3
**Ce**	0.137	0.100	0.240	102.1
	0.000	0.100	0.98	98.0
	0.216	0.200	0.420	101.9
**Pr**	< 0.011	0.020	0.0198	98.9
	< 0.011	0.020	0.0198	99.1
	< 0.011	0.020	0.0191	95.4
**Sm**	< 0.006	0.0050	0.0050	100.2
	< 0.006	0.0050	0.0049	98.5
	< 0.006	0.0050	0.0049	97.7
**Ho**	< 0.006	0.0050	0.0052	103.1
	< 0.006	0.0050	0.0050	99.1
	< 0.006	0.0050	0.0048	95.5
