# Supplementary material for: The Formation of the Elemental Composition of Young Chardonnay Wine Through Fining with Organic Fining Agents
Source: Molecules. 2026 Sep 10;31(18):3199. doi: 10.3390/molecules31183199 (PMC13608984; doi:10.3390/molecules31183199)
Supplement: Supplementary file 1 [file molecules-31-03199-s001.zip › Supplementary Table S4.pdf]

Supplementary Table S4. Classification functions obtained using discriminant analysis

Variable	Classification Functions				
	Anapa	Varvarovka	Vinnaya derevnya	Vinogradny	Gostagaevskaya
Cs	163.571	14.149	129.199	-29.699	-49.746
Cu	-1.672	3.240	1.357	3.551	3.207
Fe	-0.006	0.004	-0.007	0.015	0.004
Li	3.927	3.906	11.191	12.125	23.294
Mn	0.357	0.345	0.485	0.838	0.311
Zr	-8.621	0.856	-5.099	1.229	3.282
Constant	-557.486	-264.245	-750.420	-891.977	-456.128
