# Supplementary material for: The Formation of the Elemental Composition of Young Chardonnay Wine Through Fining with Organic Fining Agents
Source: Molecules. 2026 Sep 10;31(18):3199. doi: 10.3390/molecules31183199 (PMC13608984; doi:10.3390/molecules31183199)
Supplement: Supplementary file 1 [file molecules-31-03199-s001.zip › Supplementary Table S5.pdf]

Supplementary Table S5. Information about fining agents

Name	Country	Fining agent type	Composition	Mechanism of action / Effect	Recommended Dosage (applied dosage), mg/L	Advantages	Disadvantages
TANENOL BLANC	Italy	Organic, plant	Tanins	Elimination of heat-resistant proteins that cause turbidity; color stabilization	40–80 (50)	Prevents browning; stabilizes color	Variable efficiency; requires long contact time
PK SOL M2	Italy	Organic, plant	PVPP + pea protein + silica gel	Rapid lightening by exposure to tannins and phenolic substances; reduces the risk of browning	200–600 (250)	It acts effectively on tannins and phenols, reducing the risk of browning.	Expensive
PK SOL M4	Italy	Organic, plant	Chitosan + pea protein + silica gel	Effects on polyphenolic and oxidized fractions; elimination of protein and colloidal turbidity.	50–300 (150)	Eliminates protein and colloidal turbidity; effects on oxidized substances.	May introduce protein residues, allergy risk
CLEAR V	Italy	Organic, plant	pea protein	Efficient deposition by forming a compact sediment.	50–300 (200)	Forms a compact sediment; highly efficient	May introduce allergenic proteins
No Brett Inside	Denmark	Organic, biopolymer (fungal origin)	Chitosan (fungal origin)	Effective control of Brettanomyces bruxellensis yeast; preventing the formation of volatile phenols and "animal" aroma.	40–80 (60)	Of natural origin; prevents unpleasant "animal" odour	Expensive; possible allergy to fungal proteins
Vinogel	Russia	Organic, animal	Gelatin	Prevention of colloidal turbidity; elimination of defects in smell, taste and color.	20–50 (30)	Cheap; prevents colloidal turbidity; eliminates organoleptic defects	Non-vegetarian/vegan; contains allergens
ELECTRA	Italy	Inorganic, mineral	Bentonite clay	Clarification of wine through ion exchange	200–1000 (300)	Widespread, cheap and highly effective	Risk of aluminum contamination; removes useful components (affects color and aroma)
