# Supplementary material for: The Formation of the Elemental Composition of Young Chardonnay Wine Through Fining with Organic Fining Agents
Source: Molecules. 2026 Sep 10;31(18):3199. doi: 10.3390/molecules31183199 (PMC13608984; doi:10.3390/molecules31183199)
Supplement: Supplementary file 1 [file molecules-31-03199-s001.zip › Supplementary Table S6.pdf]

Supplementary Table S6. Operational parameters of spectrometers and limits of quantification

ICP-MS (iCAP RQ)		ICP-AES (iCAP 7400)	
Plasma gas flow-rate, L/min	15.0	Plasma gas flow-rate, L/min	12
Nebulizer gas flow-rate, L/min	1.0	Nebulizer gas flow-rate, L/min	0.5
Applied power, W	1550	Applied power, W	1200
Replicates	5	Replicates	5
Auxllary gas flow-rate, L/min	0.8	Auxllary gas flow-rate, L/min	0.5
Integration time, s	0.01		
Analytes measured, (LOQ, μg/L)	^{7}Li, (0.001)<br>^{9}Be, (0.001<br>^{49}Ti, (0.081)<br>^{51}V, (0.014)<br>^{59}Co, (0.008)<br>^{75}As, (0.015)<br>^{89}Y, (0.001)<br>^{90}Zr, (0.003)<br>^{98}Mo, (0.006)<br>^{107}Ag, (0.04)<br>^{111}Cd, (0.002)<br>^{120}Sn, (0.08)<br>^{133}Cs, (0.002)<br>^{182}W, (0.006)<br>^{208}Pb, (0.009)<br>^{140}Ce, (0.001)<br>^{163}Dy, (0.003)<br>^{166}Er, (0.003)<br>^{151}Eu, (0.003)<br>^{157}Gd, (0.002)<br>^{165}Ho, (0.006)<br>^{139}La (0.001)<br>^{175}Lu, (0.002)<br>^{146}Nd, (0.014)<br>^{141}Pr, (0.011)<br>^{152}Sm, (0.006)<br>^{159}Tb, (0.005)<br>^{169}Tm, (0.002)<br>^{174}Yb, (0.002)<br><br>Interference correction equations<br>Sm=I(^{152}Sm)-0.012780*I(^{157}Gd)<br>Yb = I(^{174}Yb)-0.005934×I(^{178}Hf)	Analytes measured, (LOQ, μg/L)	Zn 213.856 (I), (0.35)<br>Ni 231.604 (II), (0.26)<br>Mn 257.610 (II), (0.15)<br>Fe 259.940 (II), (1.6)<br>Mg 280.270 (II), (8)<br>Cu 324.754 (I), (0.11)<br>Al 396.152 (I), (1.23)<br>Sr 421.552 (II), (0.57)<br>Ca 422.673 (I), (14)<br>Ba 455.403 (II), (0.41)<br>Na 588.995 (I), (2.4)<br>K 766.490 (I), (5.0)<br>Rb 780.023 (I) (0.46)<br><br>I – neutral atom,<br>II – singly ionized atom
